# Persistent Chlamydial Infections: An In Vivo Reality or a Cell Culture Artifact?

**DOI:** 10.1155/S1064744996000324

**Published:** 1996

**Authors:** Gerald I. Byrne

**Affiliations:** Department of Medical Microbiology and Immunology University of Wisconsin School of Medicine Madison WI USA


Infectious Diseases in Obstetrics and Gynecology 4:149–151 (1996)
© 1996 Wiley-Liss, Inc.

### Persistent Chlamydial Infections: An In Vivo Reality or a Cell Culture Artifact?

Gerald I. Byrne

*Department of Medical Microbiology and Immunology, University of Wisconsin, Madison, School of Medicine, Madison, WI*

KEY WORDS

*C. trachomatis; C. pneumoniae;* chronic upper genital tract infection

*Chlamydia trachomatis* and *Chlamydia pneumoniae* are pathogens with an extremely narrow host range (humans) and are limited to existence within the confines of a membrane-bound vesicle contained in the cytoplasm of susceptible host cells. In the case of *C. trachomatis,* with the notable exception of the lymphogranuloma venereum biovar, active growth and progression to a productive infection, with generation of infectious forms (elementary bodies, EB), appears to be restricted to epithelial cells lining either ocular (conjunctiva) or genital (urethra in males, endocervix to fallopian tubes in females) mucosal surfaces, although spread to deeper tissue and in the case in Reiter's disease, synovium, is possible.[1] In the case of *C. pneumoniae,* a broader host cell range exists that includes respiratory epithelial cells, alveolar macrophages and perhaps monocytes and cells of the vascular endothelium.[2]

Long term sequelae of acute human *C. trachomatis* and *C. pneumoniae* infections include chronic local tissue damage that may reflect persistent or occult infection, alone or superimposed upon reinfection that lead to immunologic consequences which are directly associated with disease manifestations. Chronic aspects of chlamydial infections include scarring trachoma,[3] damage to fallopian tubes[4] for *C. trachomatis* and possibly development of atherosclerotic lesions[5] for *C. pneumoniae.* At present it is unclear what, if any, role persistent, viable but metabolically quiescent chlamydiae play in the more chronic aspects of human chlamydial infections, but extensive cell culture studies have provided evidence that chlamydial persistence in the form of aberrently growing organisms can be induced by a variety of external stimuli and more recently in vivo data has in large measure supported claims made based on cell culture models of persistence.

### CELL CULTURE MODELS OF PERSISTENCE

Chlamydia normally proceed through an orderly alternation of functionally and morphologically distinct developmental forms that begin and end with the production of metabolically inactive, infectious EB, interspersed with the metabolically active, but non-infectious intracellular form called the reticulate body (RB). Productive infections necessarily begin and end with EB, but a variety of cell culture manipulations can be introduced that prevent or delay RB from differentiating to EB. The result of these manipulations extend the length of time non-infectious RB remain within intracellular cytoplasmic vesicles and often this extended period of non-productive growth is associated with the development of morphologically abnormal (often very large) intracellular forms of the organism. Variations of these alternative growth options have been studied in the context of antibiotic treatments (penicillin, some quinalones), nutrient deprivation (especially amino acids), cytokine-mediated activation of host cells (also resulting in nutrient deprivation, especially tryptophan) and a variety of other stressful changes in the environment, including heat shock. Collectively, these various studies that involve production of chronic cell culture models of chlamydial growth have been referred to as persistence and have been the subject of several recent review articles.[6,7]

Persistence is a term that was developed to dis-

Received September 15, 1996
Accepted October 1, 1996

Article tinguish the usual mode of intracellular chlamydial development from the morphologically altered intracellular forms that often arise under conditions when extended RB development occurs. Clearly, the latter mode of intracellular existence should not be considered latency since a metabolically active form of the organism is continuously present, even though infectivity is not achieved since EB do not emerge in a timely manner. Cell culture persistence also is not a dead-end pathway, since productive infections can resume after the persistence-inducing conditions have been alleviated.

An interesting feature of cell culture persistence that has been associated with cytokine-mediated activation of infected host cells is a change in the pattern of protein expression that reflects a stress-related response and a decline in the expression of constitutive and developmentally-regulated envelope proteins. It has been suggested[6,7] that if similar changes in protein expression could be documented in vivo in association with chronic chlamydial disease, then this could help explain disease pathogenesis since chlamydial stress-response proteins have been implicated as mediators of untoward immune reactivity in animal models of chlamydial infections.[8]

### PERSISTENCE IN VIVO

Documentation of the details associated with persistent growth have remained restricted to cell culture models. However several in vivo investigations have provided inferential support for the hypothesis that persistent chlamydiae contribute to long term sequelae of chlamydial disease in vivo. Certain features associated with in vivo growth would be consistent with growth options that include some form of persistence. These include the presence of abnormally large intracellular forms of the organism in diseased tissue, evidence for chlamydial nucleic acid in the absence of demonstrable culturable forms, immunologic evidence for heightened reactivity to stress response proteins and the continuous presence of chlamydial antigens in the absence of re-infections.

To one degree or another, at least some of these conditions have been met for several chronic chlamydial diseases.[9] The most likely clinical situations involving the presence of persistent *C. trachomatis* include, chronic upper genital tract disease in women (chlamydial genomes identified in submucosal tissue in the absence of infectious organisms) and Reiter's disease (persistently infected synovial cells perhaps expressing stress response gene transcripts). The most likely clinical situations involving the presence of persistent *C. pneumoniae* include atherosclerotic lesions (PCR and RT-PRC evidence for genomes and transcripts) and some forms of adult onset asthma (chronic disease with serologic evidence for *C. pneumoniae*). Work using actual clinical disease to verify the involvement of persistent chlamydiae in the disease process have thus far been limited in scope and far from conclusive. Sufficient data has been generated, however, to warrant more extensive studies to support or refute the presence of occult chlamydiae as a mediator of pathogenesis.

### PROSPECTUS

Study of clinical material from various human chlamydial syndromes for evidence of persistent chlamydiae will eventually provide a definitive answer to the question of chlamydial persistence. Other work also will be of value in better defining alternative modes of intracellular chlamydial growth as these relate to the disease process. Use of appropriate animal models including the *C. trachomatis* mouse pneumonitis model for chlamydial upper genital tract disease in female mice and the ApoE-deficient mouse model for the study of cardiac involvement for *C. pneumoniae*[10] will be instructive. In addition, cell culture models that more accurately reflect in vivo conditions should help to provide details concerning the conditions under which chlamydiae are likely to enter persistent growth states. For example, in recent studies using polarized human genital epithelial cells, we have found that persistent *C. trachomatis* growth can be established and maintained much much more readily than with the more standard conditons of cell culture (Kane and Byrne, unpublished).

Application of each of these broad areas of research (evaluation of clinical samples, animal models and more appropriate mimicks of in vivo conditions) will combine to provide a clear answer to the question of whether persistent growth is an in vivo reality or a cell culture artifact.

### REFERENCES

1. Rahman MU, Cheema MA, Schumacher HR, Hudson AP: Molecular evidence for the presence of Chlamydia in the synovium of patient's with Reiter's syndrome. Arthritis Rheum. 35:521–529, 1992.

2. Gaydos CS, Summersgill JD, Sahney NN, Ramirez JA, Quinn TC: Replication of *Chlamydia pneumoniae* in vitro in human macrophages, endothelial cells and aortic artery smooth muscle cells. Infect Immun 64:1614–1620, 1996.
3. Schachter J, Dawson CR: Human Chlamydial Infections. PSG Publishing Co. Littleton, MA, 1978.
4. Sellors JW, Mahoney JB, Chernesky MA, Rath DA: Tubal factor infertility: An association with prior chlamydial infection and asymptomatic salpingitis. Fertil Steril 49:451–457, 1988.
5. Kuo CC, Jackson L, Campbell LA, Grayston JT: *Chlamydiae pneumoniae* (TWAR). Clin Microbiol Rev 8:451–461, 1995.
6. Beatty WL, Byrne GI, Morrison RP: Repeated and persistent infection with Chlamydia and the development of chronic inflammation and disease. Trends Microbiol 2:94–98, 1994.
7. Beatty WL, Morrison RP, Byrne GI: Persistent chlamydiae: From cell culture to a paradigm for chlamydial pathogenesis. Microbiol Rev 58:686–699, 1994.
8. Morrison RP, Lyng K, Caldwell HD: Chlamydial disease pathogenesis: Ocular hypersensitivity elicited by a genus-specific 57-kD protein. J Exp Med 169:663–675, 1989.
9. Stary A (ed.). Proceedings Third meeting of the European Society for Chlamydia Research. Study group for STD and Dermatological Microbiology of the Austrian Society for Dermatology and Venerology. Vienna, 1996.
10. Campbell LA, Moazed TC, Kuo CC, Grayston JT: Mouse models of atherosclerosis and *Chlamydia pneumoniae* infection. In: A Stary (ed.). Proceedings of the Third Meeting of the European Society for Chlamydia Research. 3:106, 1996.
